# About Children

**Published:** 1897-11

**Authors:** 


### Book Notices.

ABOUT CHILDREN. Six Lectures Given to the Nurses in the Training School of the Cleveland General Hospital, in February, 1896. By Samuel W. Kelley, M. D., Professor of Diseases of Children in the Cleveland College of Physicians and Surgeons, (Med. Dept. Ohio Wesleyan Univ.); Pediatrist to the Cleveland General Hospital; Consulting Physician to the Cleveland City Hospital; President 1896 and 1897, Ohio State Pediatric Society; Editor Cleveland Medica lGazette. Cleveland Medical Publishing Company, Cleveland Ohio, Publishers, 1897.

This little volume of 197 pages is not only instructive, but is written in a style so very pleasing that it is really interesting all the way through. It contains much valuable information and many practical suggestions for the proper care of children. While it represents a series of lectures delivered before a nurses' training school and is therefore particularly for nurses, it can be read with profit by all medical students and the majority of practicing physicians, in fact, by mothers and all who are interested in the care of children. We heartily commend the book and congratulate the author. S. E. H.

